# Supplementary figures and images for: A comparison of non-magnetic and magnetic beads for measuring IgG antibodies against Plasmodium vivax antigens in a multiplexed bead-based assay using Luminex technology (Bio-Plex 200 or MAGPIX)
Source: PLoS One. 2020 Dec 4;15(12):e0238010. doi: 10.1371/journal.pone.0238010 (PMC7717507; doi:10.1371/journal.pone.0238010)

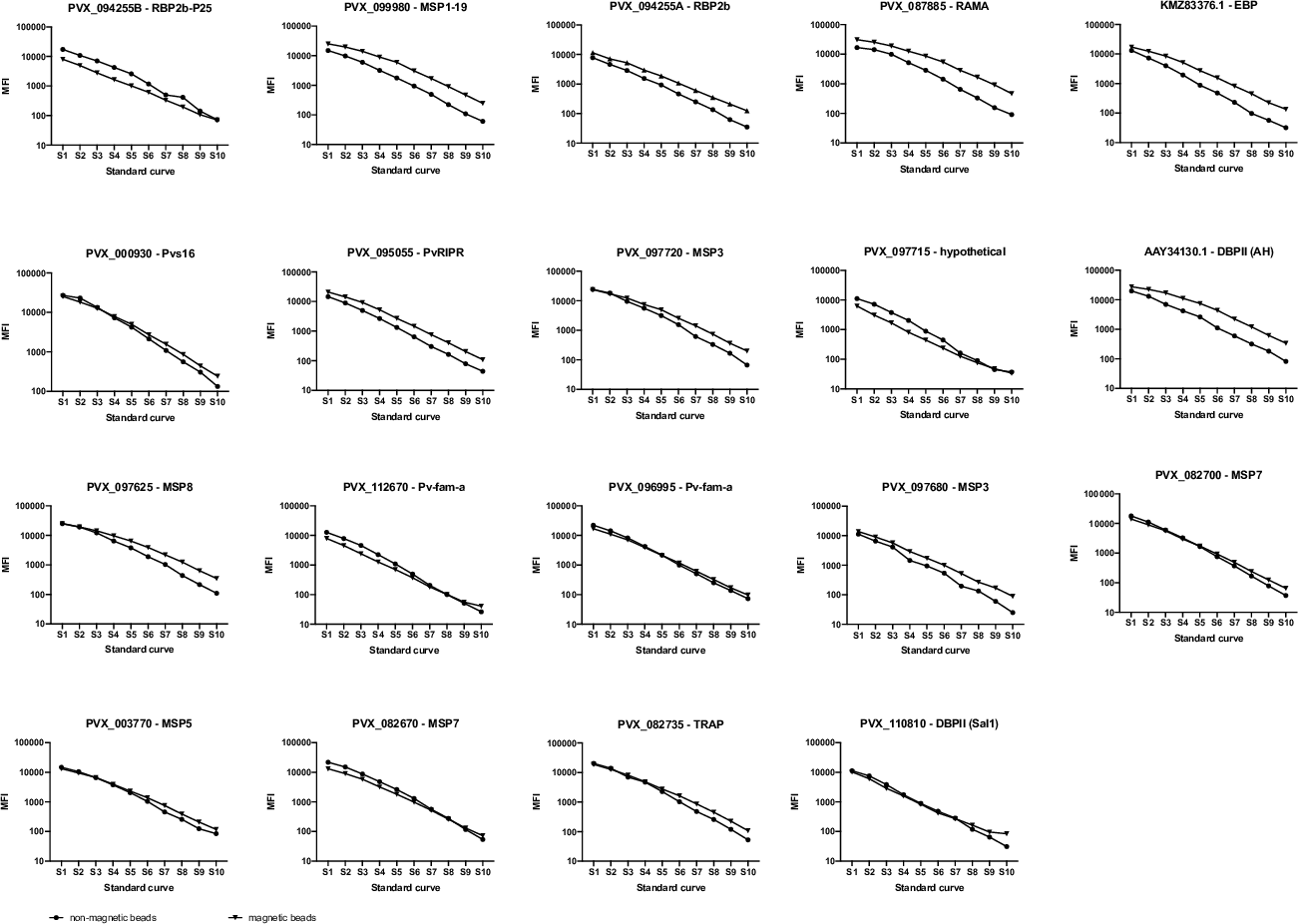

Supplement: S1 Fig — MFI = median fluorescent intensity. S1 –S10 = standard 1 to standard 12 (2 fold serial dilution of positive plasma pool, starting at 1/50 dilution). The data are converted from MFI to relative antibody units (RAU) using a five-parameter logistic function to obtain an equivalent dilution value compared to the PNG control plasma. For example, an MFI of similar value to that of the 1/50 dilution of the standard curve would result in an RAU of around 0.02 (or 1/50). The RAU values therefore range from 1.95×10^−5^ (equivalent to 1/51,200 or S11, as the curve is extrapolated one step further) to 0.02. (TIF) [file pone.0238010.s001.tif]

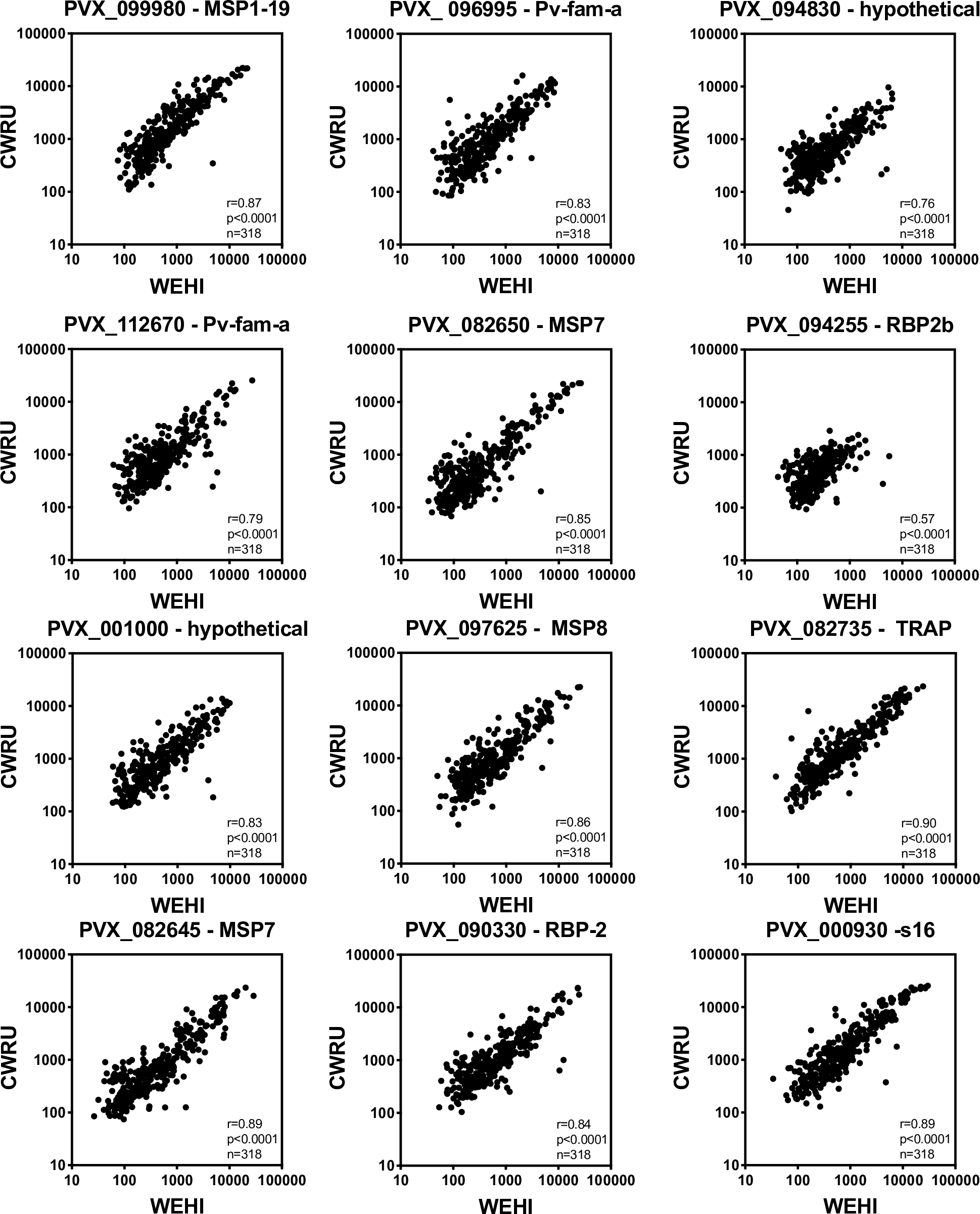

Supplement: S2 Fig — (TIF) [file pone.0238010.s002.tif]

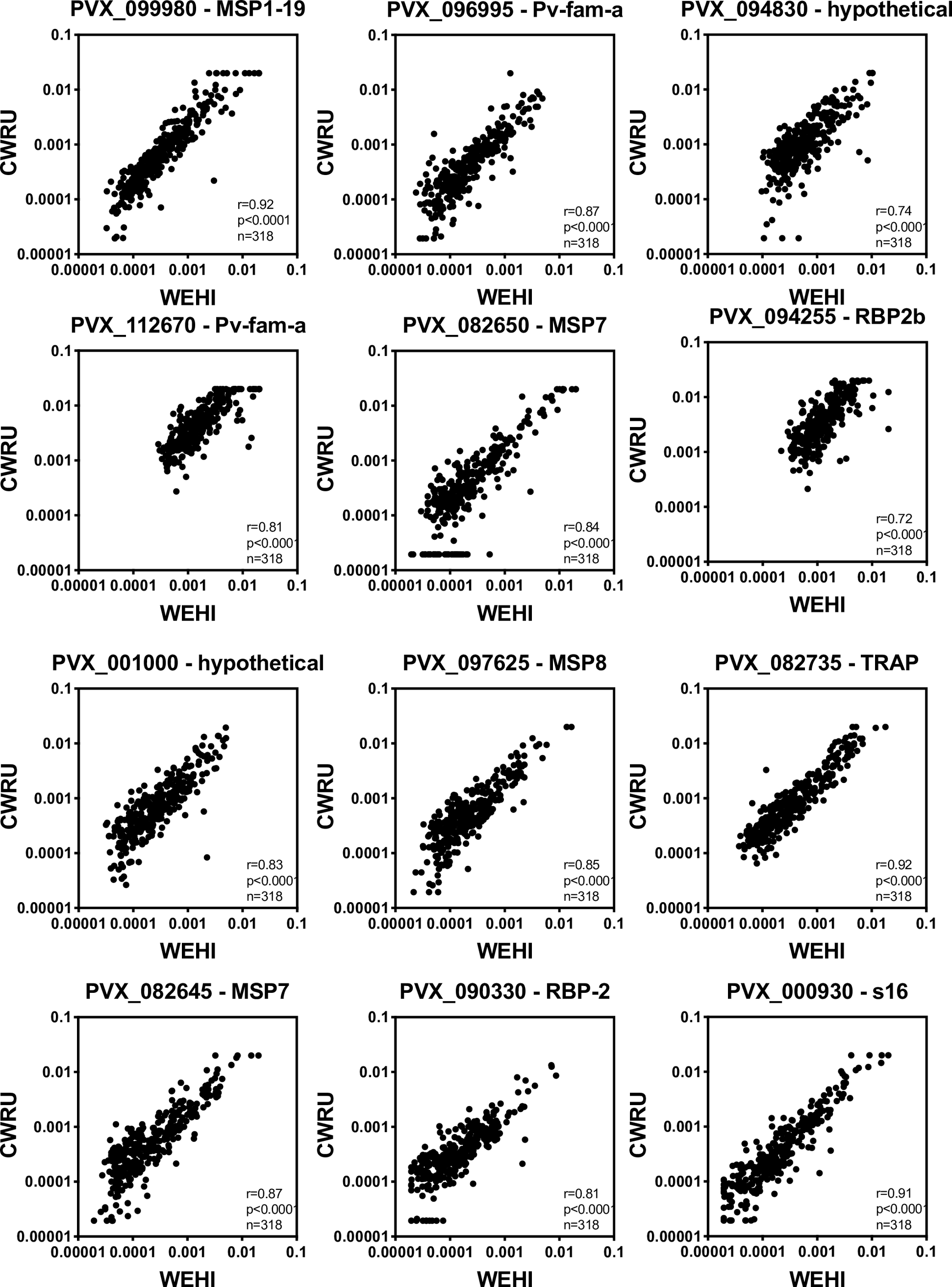

Supplement: S3 Fig — (TIF) [file pone.0238010.s003.tif]

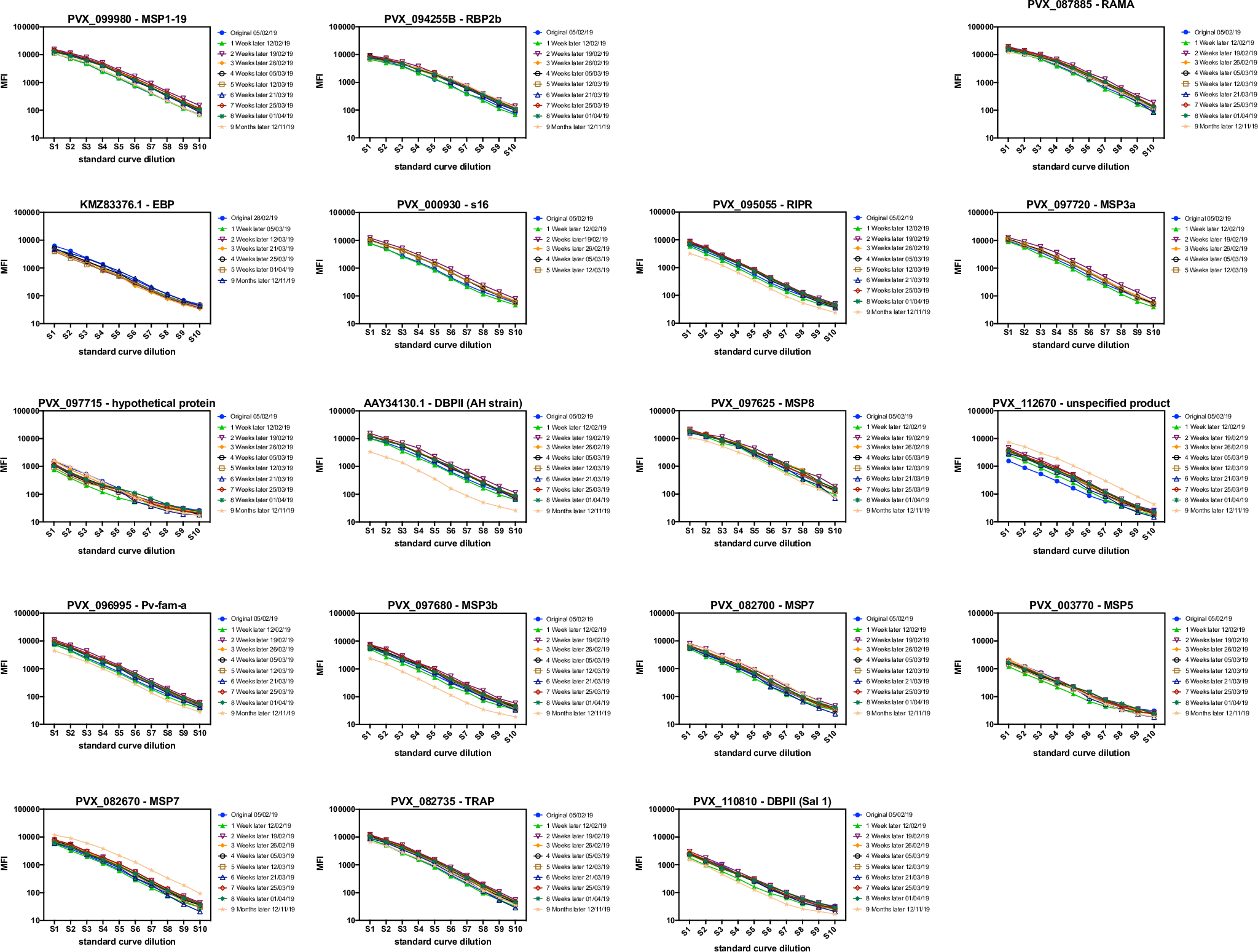

Supplement: S4 Fig — The original coupled beads were tested at every week for 2 months after coupling, then again at 9 months post-coupling. The MFI of the standard curves are presented (S1 = 1/50, then 2-fold serial dilution). New vials of secondary antibodies were opened on 19/02/19, 26/02/19 and 08/03/19. Protein PVX_094255 (WGCF construct) was not tested in this experiment. (TIF) [file pone.0238010.s004.tif]
