# Supplementary material for: A comparison of non-magnetic and magnetic beads for measuring IgG antibodies against Plasmodium vivax antigens in a multiplexed bead-based assay using Luminex technology (Bio-Plex 200 or MAGPIX)
Source: PLoS One. 2020 Dec 4;15(12):e0238010. doi: 10.1371/journal.pone.0238010 (PMC7717507; doi:10.1371/journal.pone.0238010)
Supplement: Attachment — Submitted filename: ResponsetoReviewers_16Nov2020.docx [file pone.0238010.s006.docx]

Response to Reviewers

Reviewer #2: The authors have made changes in the manuscript that enhance its clarity and addressed the issues raised by the reviewers. The study clearly makes the point that a correlation exists among the data generated using difference beads-based systems. The authors might consider the following suggestions:
1. The authors added the sentence: “This is expected given the conversion, based on the standard curve generated with a plasma pool from immune PNG donors…..” Was the standard curve run on each plate? If so, please add this information to the text.

We have updated the sentence to read “This is expected given the conversion, based on the standard curve generated with a plasma pool from immune PNG donors that is run on every plate, is used to account for any plate-plate variation and potentially to overcome differences that might be attributed to different machines maintained and set-up with different calibration and validation bead sets.

We also updated the methods text to make it clear that the standard curve is run on every plate.

2. Supporting Fig. 1 shows the linearity of the dilution curve. However, it is not totally clear how one obtains relative arbitrary unit (RAU) from the curve (MFI vs S1, S2, etc.). Could the authors please add information to the figure legend on how to convert the data from MFI to RAU?

We have added the following text supporting Fig. 1 legend:

The data are converted from MFI to relative antibody units (RAU) using a five-parameter logistic function to obtain an equivalent dilution value compared to the PNG control plasma. For example, an MFI of similar value to that of the 1/50 dilution of the standard curve would result in an RAU of around 0.02 (or 1/50). The RAU values therefore range from 1.95×10^−5^ (equivalent to 1/51,200 or S11, as the curve is extrapolated one step further) to 0.02.

3. Supportive Fig. 2 – YEAH! This figure shows the raw MFI and that subsequent statistical refinements not really needed. All readers will appreciate this figure.

Thank you for your positive response!

4. Supportive Fig. 3 – shows the relationship of the adjusted values and documents the association after statistical adjustments. Interestingly, adjustments changed the correlations insignificantly. Nice to see the comparison.

Thank you for your positive response!

5. Supplemental Figures 3 and 4 appear to be the same – comparison of RAU at CWRU and WHEI. Maybe the wrong figure was uploaded into the copy I received. Supportive Figure 4 doesn’t show changes in MFI over time. The authors need to check sFig. 4.

Thank you for noticing this, we have replaced sFig. 4. with the correct figure.
